# 8-Fluoro-4-oxo-4*H*-chromene-3-carbalde­hyde

**DOI:** 10.1107/S1600536814013208

**Published:** 2014-06-14

**Authors:** Yoshinobu Ishikawa

**Affiliations:** a School of Pharmaceutical Sciences, University of Shizuoka, 52-1 Yada, Suruga-ku, Shizuoka 422-8526, Japan

## Abstract

In the title compound, C_10_H_5_FO_3_, the non-H atoms of the 8-fluoro­chromone unit are essentially coplanar (r.m.s. deviation = 0.0259 Å), with a largest deviation from the mean plane of 0.0660 (12) Å for the chromone carbonyl O atom. The formyl group is twisted with respect to the attached ring [C—C—C—O torsion angles = −11.00 (19) and 170.81 (11)°]. In the crystal, mol­ecules are linked *via* weak C—H⋯O hydrogen bonds along the *a* axis and [-101], forming corrugated layers parallel to (010). In addition, π–π stacking inter­actions [centroid–centroid distance between the planes of the pyran and benzene rings = 3.519 (2) Å] are observed between these layers.

## Related literature   

For related structures, see: Ishikawa & Motohashi (2013 ▶); Ishikawa (2014 ▶). For the synthesis of the precursor of the title compound, see: Valoti *et al.* (2001 ▶). For halogen bonding, see: Auffinger *et al.* (2004 ▶); Metrangolo *et al.* (2005 ▶); Wilcken *et al.* (2013 ▶); Sirimulla *et al.* (2013 ▶).
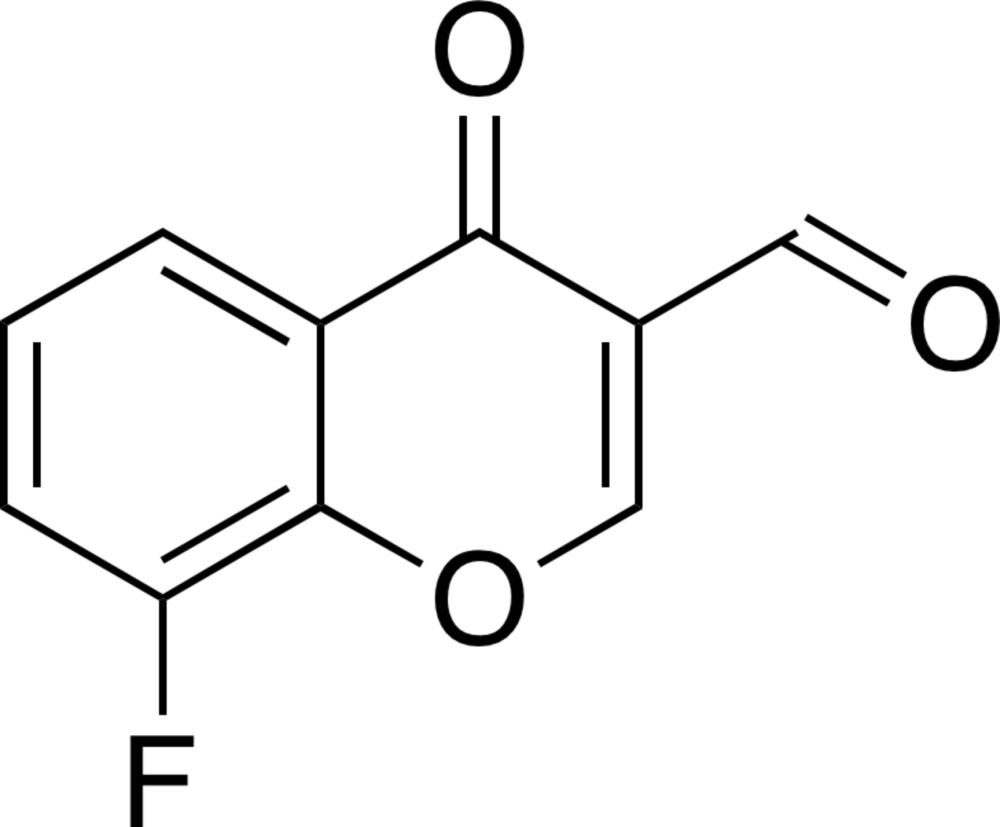


## Experimental   

### 

#### Crystal data  

C_10_H_5_FO_3_
*M* *_r_* = 192.15
Monoclinic, 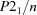

*a* = 6.6643 (12) Å
*b* = 8.395 (5) Å
*c* = 14.247 (4) Å
β = 97.865 (16)°
*V* = 789.6 (6) Å^3^
*Z* = 4
Mo *K*α radiation
μ = 0.14 mm^−1^
*T* = 100 K
0.40 × 0.40 × 0.20 mm

#### Data collection  

Rigaku AFC-7R diffractometer
2445 measured reflections
1810 independent reflections
1606 reflections with *F* ^2^ > 2σ(*F* ^2^)
*R*_int_ = 0.049
3 standard reflections every 150 reflections intensity decay: −0.4%

#### Refinement  

*R*[*F* ^2^ > 2σ(*F* ^2^)] = 0.038
*wR*(*F* ^2^) = 0.109
*S* = 1.07
1810 reflections
127 parameters
H-atom parameters constrained
Δρ_max_ = 0.28 e Å^−3^
Δρ_min_ = −0.36 e Å^−3^

### 

Data collection: *WinAFC Diffractometer Control Software* (Rigaku, 1999 ▶); cell refinement: *WinAFC Diffractometer Control Software*; data reduction: *WinAFC Diffractometer Control Software*; program(s) used to solve structure: *SIR2008* (Burla, *et al.*, 2007 ▶); program(s) used to refine structure: *SHELXL97* (Sheldrick, 2008 ▶); molecular graphics: *CrystalStructure* (Rigaku, 2010 ▶); software used to prepare material for publication: *CrystalStructure*.

## Supplementary Material

Crystal structure: contains datablock(s) General, I. DOI: 10.1107/S1600536814013208/lh5713sup1.cif

Structure factors: contains datablock(s) I. DOI: 10.1107/S1600536814013208/lh5713Isup2.hkl

Supporting information file. DOI: 10.1107/S1600536814013208/lh5713Isup3.cml

CCDC reference: 1007014

Additional supporting information:  crystallographic information; 3D view; checkCIF report

## Figures and Tables

**Table 1:** Hydrogen-bond geometry (Å, °)

*D*—H⋯*A*	*D*—H	H⋯*A*	*D*⋯*A*	*D*—H⋯*A*
C1^i^—H1^i^⋯O2	0.95	2.48	3.285 (2)	142 (1)
C6^ii^—H4^ii^⋯O2	0.95	2.41	3.221 (2)	143 (1)

Symmetry codes: (i) 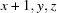; (ii) 
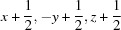
.

**Fig. 1.**
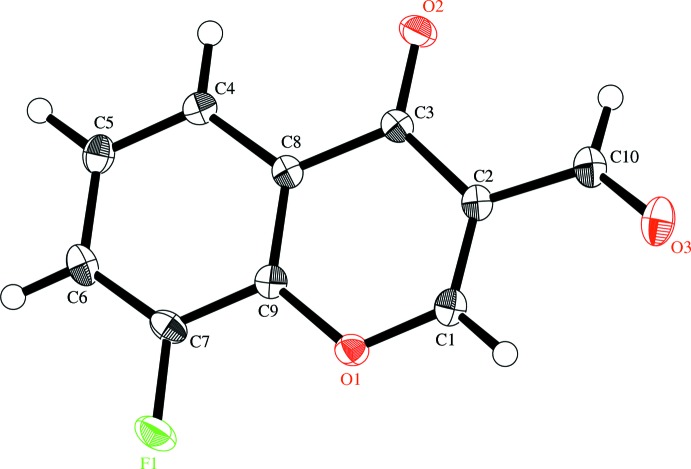
The molecular structure of the title compound with displacement ellipsoids drawn at the 50% probability level. Hydrogen atoms are shown as small spheres of arbitrary radius.

**Fig. 2.**
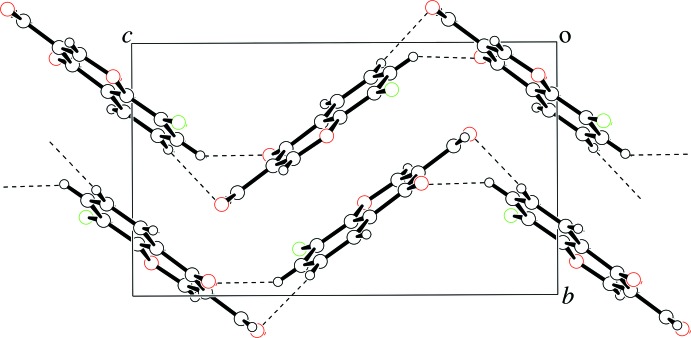
A crystal packing view of the title compound. Intermolecular C–H···O hydrogen bonds are represented by dashed lines.

**Fig. 3.**
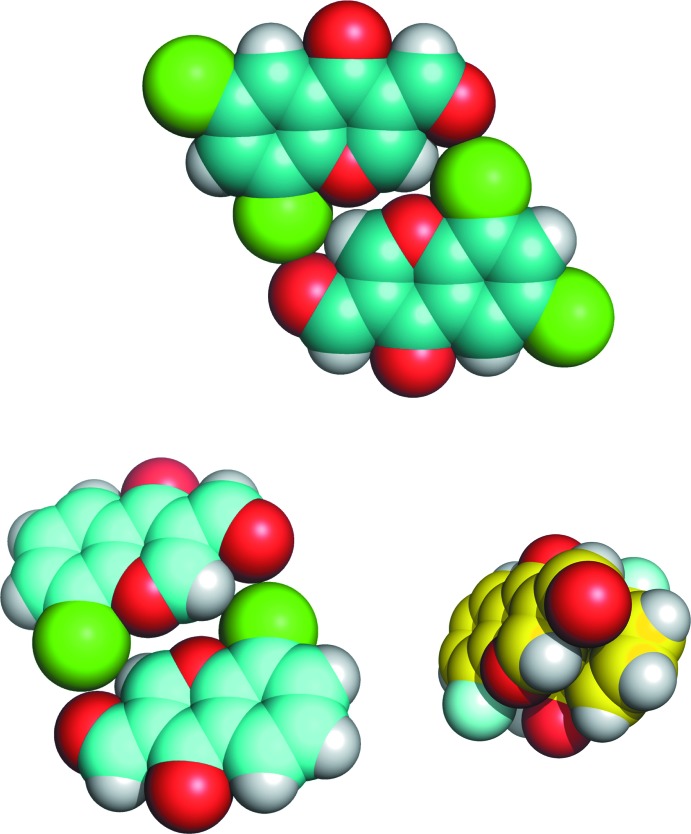
Sphere models of the crystal structures of 6,8-dichloro-4-oxochromene-3-carbaldehyde (top), 8-chloro-4-oxo-4H-chromene-3-carbaldehyde (bottom left), and the title compound (bottom right).

## supplementary crystallographic information

### S1. Comment

Halogen bonds have been found to occur in organic, inorganic, and biological systems, and have recently attracted much attention in medicinal chemistry, chemical biology and supramolecular chemistry (Auffinger *et al.*, 2004, Metrangolo *et al.*, 2005, Wilcken *et al.*, 2013, Sirimulla *et al.*, 2013). We have recently reported the crystal structures of a dichlorinated 3-formylchromone derivative 6,8-dichloro-4-oxochromene-3-carbaldehyde (Ishikawa & Motohashi, 2013, Fig. 3 (top)) and a monochlorinated one 8-chloro-4-oxo-4*H*-chromene-3-carbaldehyde (Ishikawa, 2014, Fig. 3 (bottom left)). It was found that halogen bonding is observed for 6,8-dichloro-4-oxochromene-3-carbaldehyde between the formyl oxygen atom and the chlorine atom at 8-position, but is not observed for 8-chloro-4-oxo-4*H*-chromene-3-carbaldehyde between the formyl oxygen atom and the chlorine atom at 8-position. As part of our interest in this type of chemical bonding, we herein report the crystal structure of a monofluorinated 3-formylchromone derivative 8-fluoro-4-oxo-4*H*-chromene-3-carbaldehyde. The objective of this study is to reveal whether halogen bond(*s*) can be formed in the crystal structure of this compound with the fluorine atom at 8-position.

The mean deviation of the least-square planes for the non-hydrogen atoms of the 8-fluorochromone unit is 0.0259 Å, and the largest deviations is 0.0660 (12) Å for O2 (Fig. 1). These mean that these atoms are essentially coplanar. The formyl group is twisted [C1–C2–C10–O3 = -11.00 (19)° and C3–C2–C10–O3 = 170.81 (11)°]. In the crystal, the molecules are linked *via* C–H···O hydrogen bonds along the *a* axis and (1,0,1) to form corrugated layers parallel to (010) and *via*π–π stacking interaction [*C*g–*C*g distance between the pyran and benzene rings = 3.519 (2) Å] of different layers (Fig. 2). The distance between the fluorine atom and the oxygen atom of the chromone carbonyl group [3.332 (3) Å] are far from halogen bonding (Fig.3 (bottom right)).

### S2. Experimental

2-Hydroxy-3-fluoroacetophenone was prepared according to the literature method (Valoti *et al.* 2001). To a solution of 2-hydroxy-3-fluoroacetophenone (7.1 mmol) in *N*,*N*-dimethylformamide (15 ml) was added dropwise POCl_3_ (17.7 mmol) at 273 K. After the mixture was stirred for 14 h at room temperature, water (50 ml) was added. The precipitates were collected, washed with water, and dried *in vacuo* (yield: 57%). ^1^H NMR (400 MHz, DMSO-*d*_6_): *δ* = 7.58 (m, 1H), 7.88 (dd, 1H, *J* = 1.5 and 8.3 Hz), 7.95 (d, 1H, *J* = 8.3 Hz), 8.99 (s, 1H), 10.13 (s, 1H). DART-MS calcd for [C_10_H_5_F_1_O_3_ + H^+^]: 193.030, found 193.035. Single crystals suitable for X-ray diffraction were obtained by slow evaporation of a 2-butanone solution of the title compound at room temperature.

### S3. Refinement

The C(*sp*^2^)-bound hydrogen atoms were placed in geometrical positions [C–H 0.95 Å, *U*_iso_(H) = 1.2*U*_eq_(C)], and refined using a riding model.

### Crystal data

**Table:**

C_10_H_5_FO_3_	*F*(000) = 392.00
*M**_r_* = 192.15	*D*_x_ = 1.616 Mg m^−^^3^
Monoclinic, *P*2_1_/*n*	Mo *K*α radiation, λ = 0.71069 Å
Hall symbol: -P 2yn	Cell parameters from 25 reflections
*a* = 6.6643 (12) Å	θ = 15.5–17.4°
*b* = 8.395 (5) Å	µ = 0.14 mm^−^^1^
*c* = 14.247 (4) Å	*T* = 100 K
β = 97.865 (16)°	Block, yellow
*V* = 789.6 (6) Å^3^	0.40 × 0.40 × 0.20 mm
*Z* = 4	

### Data collection

**Table:**

Rigaku AFC-7R diffractometer	θ_max_ = 27.5°
ω–2θ scans	*h* = −8→8
2445 measured reflections	*k* = −6→10
1810 independent reflections	*l* = −10→18
1606 reflections with *F*^2^ > 2σ(*F*^2^)	3 standard reflections every 150 reflections
*R*_int_ = 0.049	intensity decay: −0.4%

### Refinement

**Table:**

Refinement on *F*^2^	Secondary atom site location: difference Fourier map
*R*[*F*^2^ > 2σ(*F*^2^)] = 0.038	Hydrogen site location: inferred from neighbouring sites
*wR*(*F*^2^) = 0.109	H-atom parameters constrained
*S* = 1.07	*w* = 1/[σ^2^(*F*_o_^2^) + (0.0617*P*)^2^ + 0.3187*P*] where *P* = (*F*_o_^2^ + 2*F*_c_^2^)/3
1810 reflections	(Δ/σ)_max_ < 0.001
127 parameters	Δρ_max_ = 0.28 e Å^−^^3^
0 restraints	Δρ_min_ = −0.36 e Å^−^^3^
Primary atom site location: structure-invariant direct methods	

### Special details

**Table:**

Refinement. Refinement was performed using all reflections. The weighted *R*-factor (*wR*) and goodness of fit (*S*) are based on *F*^2^. *R*-factor (gt) are based on *F*. The threshold expression of *F*^2^ > 2.0 σ(*F*^2^) is used only for calculating *R*-factor (gt).

### Fractional atomic coordinates and isotropic or equivalent isotropic displacement parameters (Å^2^)

**Table:**

	*x*	*y*	*z*	*U*_iso_*/*U*_eq_	
F1	0.63662 (11)	0.31393 (11)	−0.11055 (6)	0.0262 (3)	
O1	0.70736 (13)	0.13166 (11)	0.04296 (6)	0.0175 (3)	
O2	1.28166 (13)	0.05710 (12)	0.17921 (6)	0.0211 (3)	
O3	0.80302 (16)	−0.13767 (12)	0.29126 (7)	0.0265 (3)	
C1	0.74226 (18)	0.04263 (15)	0.12185 (8)	0.0174 (3)	
C2	0.92737 (18)	0.01472 (14)	0.17064 (8)	0.0156 (3)	
C3	1.10896 (18)	0.08367 (14)	0.14003 (8)	0.0148 (3)	
C4	1.22460 (18)	0.26974 (15)	0.01980 (9)	0.0169 (3)	
C5	1.18179 (19)	0.36449 (15)	−0.05972 (9)	0.0190 (3)	
C6	0.9826 (2)	0.38000 (15)	−0.10529 (9)	0.0193 (3)	
C7	0.83046 (19)	0.30073 (15)	−0.06889 (9)	0.0180 (3)	
C8	1.06800 (17)	0.18914 (14)	0.05669 (8)	0.0146 (3)	
C9	0.86964 (18)	0.20612 (14)	0.01198 (8)	0.0152 (3)	
C10	0.9457 (2)	−0.09092 (15)	0.25433 (9)	0.0197 (3)	
H1	0.6287	−0.0040	0.1450	0.0209*	
H2	1.3604	0.2591	0.0496	0.0203*	
H3	1.2884	0.4197	−0.0837	0.0228*	
H4	0.9534	0.4442	−0.1604	0.0231*	
H5	1.0775	−0.1243	0.2809	0.0236*	

### Atomic displacement parameters (Å^2^)

**Table:**

	*U*^11^	*U*^22^	*U*^33^	*U*^12^	*U*^13^	*U*^23^
F1	0.0193 (4)	0.0333 (5)	0.0246 (5)	0.0075 (4)	−0.0019 (3)	0.0064 (4)
O1	0.0142 (4)	0.0207 (5)	0.0178 (5)	0.0016 (4)	0.0028 (4)	0.0012 (4)
O2	0.0171 (5)	0.0282 (5)	0.0173 (5)	0.0014 (4)	−0.0006 (4)	0.0027 (4)
O3	0.0355 (6)	0.0226 (5)	0.0239 (5)	−0.0023 (4)	0.0130 (4)	0.0020 (4)
C1	0.0197 (6)	0.0163 (6)	0.0175 (6)	0.0008 (5)	0.0071 (5)	−0.0010 (5)
C2	0.0198 (6)	0.0138 (6)	0.0140 (6)	0.0014 (5)	0.0048 (5)	−0.0016 (5)
C3	0.0179 (6)	0.0146 (6)	0.0121 (5)	0.0018 (5)	0.0025 (4)	−0.0028 (4)
C4	0.0164 (6)	0.0171 (6)	0.0175 (6)	0.0005 (5)	0.0033 (5)	−0.0017 (5)
C5	0.0224 (6)	0.0169 (6)	0.0192 (6)	−0.0001 (5)	0.0077 (5)	0.0000 (5)
C6	0.0277 (7)	0.0164 (6)	0.0144 (6)	0.0051 (5)	0.0048 (5)	0.0006 (5)
C7	0.0187 (6)	0.0189 (6)	0.0158 (6)	0.0062 (5)	−0.0003 (5)	−0.0016 (5)
C8	0.0176 (6)	0.0133 (6)	0.0130 (5)	0.0017 (5)	0.0029 (5)	−0.0020 (5)
C9	0.0167 (6)	0.0140 (6)	0.0157 (6)	0.0020 (5)	0.0045 (5)	−0.0020 (5)
C10	0.0275 (7)	0.0166 (6)	0.0156 (6)	0.0006 (5)	0.0050 (5)	−0.0014 (5)

### Geometric parameters (Å, º)

**Table:**

F1—C7	1.3504 (15)	C4—C8	1.4048 (18)
O1—C1	1.3433 (15)	C5—C6	1.4017 (18)
O1—C9	1.3733 (16)	C6—C7	1.372 (2)
O2—C3	1.2289 (15)	C7—C9	1.3943 (18)
O3—C10	1.2133 (18)	C8—C9	1.3940 (16)
C1—C2	1.3510 (17)	C1—H1	0.950
C2—C3	1.4609 (18)	C4—H2	0.950
C2—C10	1.4776 (18)	C5—H3	0.950
C3—C8	1.4764 (17)	C6—H4	0.950
C4—C5	1.3817 (18)	C10—H5	0.950
			
F1···O1	2.6580 (14)	C8···H3	3.2724
O1···C3	2.8679 (15)	C9···H1	3.1799
O2···C1	3.5778 (16)	C9···H2	3.2714
O2···C4	2.8728 (18)	C9···H4	3.2729
O2···C10	2.8910 (18)	C10···H1	2.5538
O3···C1	2.8307 (17)	H1···H5	3.4830
C1···C7	3.586 (2)	H2···H3	2.3255
C1···C8	2.7638 (18)	H3···H4	2.3559
C2···C9	2.7571 (18)	F1···H1^xiii^	3.1465
C4···C7	2.7668 (18)	F1···H2^iii^	3.1555
C5···C9	2.7785 (19)	F1···H3^iii^	2.5618
C6···C8	2.8031 (19)	F1···H3^x^	3.5405
F1···O2^i^	3.332 (3)	F1···H5^ii^	3.0285
F1···O2^ii^	3.4454 (15)	O1···H1^xiii^	3.4194
F1···C2^ii^	3.5415 (16)	O1···H2^iii^	2.5611
F1···C4^iii^	3.5386 (17)	O1···H2^i^	3.5413
F1···C5^iii^	3.2393 (17)	O2···H1^vi^	2.4819
F1···C10^ii^	3.1720 (19)	O2···H4^v^	2.4119
O1···O2^i^	3.5495 (16)	O2···H5^vii^	2.8654
O1···O3^iv^	3.0624 (16)	O3···H1^viii^	3.2215
O1···C3^i^	3.5265 (18)	O3···H2^xii^	3.0871
O1···C4^iii^	3.3931 (16)	O3···H3^ix^	2.5650
O1···C4^i^	3.532 (3)	O3···H4^ix^	2.9967
O1···C8^i^	3.477 (2)	C1···H2^iii^	3.1811
O2···F1^i^	3.332 (3)	C1···H2^i^	3.5205
O2···F1^v^	3.4454 (15)	C3···H1^vi^	3.5325
O2···O1^i^	3.5495 (16)	C3···H4^v^	3.4094
O2···C1^vi^	3.2850 (17)	C3···H5^vii^	3.3166
O2···C6^v^	3.2207 (17)	C4···H1^i^	3.4743
O2···C7^i^	3.425 (3)	C4···H4^x^	3.4405
O2···C9^i^	3.5451 (18)	C4···H5^vii^	3.0932
O2···C10^vii^	3.530 (3)	C5···H1^i^	3.5564
O3···O1^viii^	3.0624 (16)	C6···H5^i^	3.2790
O3···C1^viii^	2.988 (3)	C6···H5^ii^	3.5954
O3···C2^viii^	3.376 (3)	C7···H3^x^	3.3670
O3···C5^ix^	3.300 (2)	C7···H5^i^	3.4926
O3···C6^ix^	3.503 (2)	C8···H4^x^	3.4258
O3···C9^viii^	3.4329 (18)	C8···H5^vii^	3.4483
C1···O2^iii^	3.2850 (17)	C9···H2^iii^	3.5355
C1···O3^iv^	2.988 (3)	C9···H3^x^	3.5098
C1···C4^i^	3.335 (3)	C10···H2^xii^	3.1732
C1···C5^i^	3.584 (3)	C10···H3^ix^	3.0252
C1···C8^i^	3.569 (2)	C10···H4^i^	3.3595
C2···F1^v^	3.5415 (16)	H1···F1^xiii^	3.1465
C2···O3^iv^	3.376 (3)	H1···O1^xiii^	3.4194
C2···C5^i^	3.584 (3)	H1···O2^iii^	2.4819
C2···C6^i^	3.516 (3)	H1···O3^iv^	3.2215
C2···C7^i^	3.516 (3)	H1···C3^iii^	3.5325
C3···O1^i^	3.5265 (18)	H1···C4^i^	3.4743
C3···C7^i^	3.423 (3)	H1···C5^i^	3.5564
C3···C9^i^	3.273 (2)	H1···H2^iii^	3.0424
C4···F1^vi^	3.5386 (17)	H1···H2^i^	3.5115
C4···O1^vi^	3.3931 (16)	H1···H4^ix^	3.1928
C4···O1^i^	3.532 (3)	H2···F1^vi^	3.1555
C4···C1^i^	3.335 (3)	H2···O1^vi^	2.5611
C4···C6^x^	3.535 (3)	H2···O1^i^	3.5413
C5···F1^vi^	3.2393 (17)	H2···O3^vii^	3.0871
C5···O3^xi^	3.300 (2)	H2···C1^vi^	3.1811
C5···C1^i^	3.584 (3)	H2···C1^i^	3.5205
C5···C2^i^	3.584 (3)	H2···C9^vi^	3.5355
C5···C6^x^	3.469 (2)	H2···C10^vii^	3.1732
C5···C7^x^	3.362 (3)	H2···H1^vi^	3.0424
C6···O2^ii^	3.2207 (17)	H2···H1^i^	3.5115
C6···O3^xi^	3.503 (2)	H2···H3^xiv^	3.5607
C6···C2^i^	3.516 (3)	H2···H5^vii^	2.5859
C6···C4^x^	3.535 (3)	H3···F1^vi^	2.5618
C6···C5^x^	3.469 (2)	H3···F1^x^	3.5405
C6···C6^x^	3.595 (2)	H3···O3^xi^	2.5650
C6···C10^i^	3.302 (3)	H3···C7^x^	3.3670
C7···O2^i^	3.425 (3)	H3···C9^x^	3.5098
C7···C2^i^	3.516 (3)	H3···C10^xi^	3.0252
C7···C3^i^	3.423 (3)	H3···H2^xiv^	3.5607
C7···C5^x^	3.362 (3)	H3···H5^xi^	3.3769
C8···O1^i^	3.477 (2)	H4···O2^ii^	2.4119
C8···C1^i^	3.569 (2)	H4···O3^xi^	2.9967
C8···C9^i^	3.500 (3)	H4···C3^ii^	3.4094
C9···O2^i^	3.5451 (18)	H4···C4^x^	3.4405
C9···O3^iv^	3.4329 (18)	H4···C8^x^	3.4258
C9···C3^i^	3.273 (2)	H4···C10^i^	3.3595
C9···C8^i^	3.500 (3)	H4···H1^xi^	3.1928
C10···F1^v^	3.1720 (19)	H4···H5^i^	3.1787
C10···O2^xii^	3.530 (3)	H4···H5^ii^	2.9478
C10···C6^i^	3.302 (3)	H5···F1^v^	3.0285
F1···H4	2.5628	H5···O2^xii^	2.8654
O2···H2	2.6123	H5···C3^xii^	3.3166
O2···H5	2.6094	H5···C4^xii^	3.0932
O3···H1	2.5079	H5···C6^i^	3.2790
C1···H5	3.2720	H5···C6^v^	3.5954
C3···H1	3.2941	H5···C7^i^	3.4926
C3···H2	2.6881	H5···C8^xii^	3.4483
C3···H5	2.6900	H5···H2^xii^	2.5859
C4···H4	3.2758	H5···H3^ix^	3.3769
C6···H2	3.2742	H5···H4^i^	3.1787
C7···H3	3.2451	H5···H4^v^	2.9478
			
C1—O1—C9	117.93 (10)	C4—C8—C9	119.10 (11)
O1—C1—C2	124.64 (12)	O1—C9—C7	117.27 (11)
C1—C2—C3	120.87 (11)	O1—C9—C8	123.09 (11)
C1—C2—C10	119.22 (12)	C7—C9—C8	119.64 (12)
C3—C2—C10	119.88 (11)	O3—C10—C2	124.11 (12)
O2—C3—C2	123.72 (11)	O1—C1—H1	117.679
O2—C3—C8	122.19 (12)	C2—C1—H1	117.685
C2—C3—C8	114.09 (10)	C5—C4—H2	119.881
C5—C4—C8	120.23 (11)	C8—C4—H2	119.890
C4—C5—C6	120.63 (12)	C4—C5—H3	119.685
C5—C6—C7	118.80 (12)	C6—C5—H3	119.687
F1—C7—C6	120.39 (12)	C5—C6—H4	120.605
F1—C7—C9	118.01 (12)	C7—C6—H4	120.598
C6—C7—C9	121.60 (12)	O3—C10—H5	117.944
C3—C8—C4	121.65 (11)	C2—C10—H5	117.943
C3—C8—C9	119.23 (11)		
			
C1—O1—C9—C7	−178.72 (10)	C8—C4—C5—C6	−0.79 (19)
C1—O1—C9—C8	2.18 (16)	C8—C4—C5—H3	179.2
C9—O1—C1—C2	−3.31 (17)	H2—C4—C5—C6	179.2
C9—O1—C1—H1	176.7	H2—C4—C5—H3	−0.8
O1—C1—C2—C3	0.63 (19)	H2—C4—C8—C3	−1.6
O1—C1—C2—C10	−177.55 (10)	H2—C4—C8—C9	−180.0
H1—C1—C2—C3	−179.4	C4—C5—C6—C7	0.70 (19)
H1—C1—C2—C10	2.5	C4—C5—C6—H4	−179.3
C1—C2—C3—O2	−176.84 (11)	H3—C5—C6—C7	−179.3
C1—C2—C3—C8	2.95 (16)	H3—C5—C6—H4	0.7
C1—C2—C10—O3	−11.00 (19)	C5—C6—C7—F1	179.50 (11)
C1—C2—C10—H5	169.0	C5—C6—C7—C9	0.15 (19)
C3—C2—C10—O3	170.81 (11)	H4—C6—C7—F1	−0.5
C3—C2—C10—H5	−9.2	H4—C6—C7—C9	−179.9
C10—C2—C3—O2	1.32 (18)	F1—C7—C9—O1	0.61 (17)
C10—C2—C3—C8	−178.89 (10)	F1—C7—C9—C8	179.74 (10)
O2—C3—C8—C4	−2.43 (18)	C6—C7—C9—O1	179.97 (11)
O2—C3—C8—C9	175.92 (10)	C6—C7—C9—C8	−0.90 (18)
C2—C3—C8—C4	177.77 (10)	C3—C8—C9—O1	1.48 (17)
C2—C3—C8—C9	−3.87 (15)	C3—C8—C9—C7	−177.60 (10)
C5—C4—C8—C3	178.39 (11)	C4—C8—C9—O1	179.88 (10)
C5—C4—C8—C9	0.03 (18)	C4—C8—C9—C7	0.80 (17)

Symmetry codes: (i) −*x*+2, −*y*, −*z*; (ii) *x*−1/2, −*y*+1/2, *z*−1/2; (iii) *x*−1, *y*, *z*; (iv) −*x*+3/2, *y*+1/2, −*z*+1/2; (v) *x*+1/2, −*y*+1/2, *z*+1/2; (vi) *x*+1, *y*, *z*; (vii) −*x*+5/2, *y*+1/2, −*z*+1/2; (viii) −*x*+3/2, *y*−1/2, −*z*+1/2; (ix) *x*−1/2, −*y*+1/2, *z*+1/2; (x) −*x*+2, −*y*+1, −*z*; (xi) *x*+1/2, −*y*+1/2, *z*−1/2; (xii) −*x*+5/2, *y*−1/2, −*z*+1/2; (xiii) −*x*+1, −*y*, −*z*; (xiv) −*x*+3, −*y*+1, −*z*.

### Hydrogen-bond geometry (Å, º)

**Table:**

*D*—H···*A*	*D*—H	H···*A*	*D*···*A*	*D*—H···*A*
C1^vi^—H1^vi^···O2	0.95	2.48	3.285 (2)	142 (1)
C6^v^—H4^v^···O2	0.95	2.41	3.221 (2)	143 (1)

Symmetry codes: (v) *x*+1/2, −*y*+1/2, *z*+1/2; (vi) *x*+1, *y*, *z*.
